# Supplementary material for: The nature of genetic and environmental susceptibility to multiple sclerosis
Source: PLoS One. 2021 Mar 22;16(3):e0246157. doi: 10.1371/journal.pone.0246157 (PMC7984655; doi:10.1371/journal.pone.0246157)
Supplement: S1 File — (PDF) [file pone.0246157.s001.pdf]

Supplemental Material

1. *Penetrance Adjustment for the Shared Environment of MZ-twins………………………………… p. 2*

   1.1. Penetrance Adjustments different Population Partitions……….…………….……… p. 2

   1.1a. Penetrance Adjustments for the (H+/ H−) Partition……………………………….…… p. 3

   1.1b. Penetrance Adjustments for the (F/ M) Partition.. p. 3

2. *Enrichment and Non-enrichment of Genotypes- Partitioning (G) into 2 Subsets – (G1) & (G2)*

   2a. The 1st Enrichment.. p. 4

   2a. The 2nd Enrichment... p. 5

   2c. Combining the Enrichment Results.. p. 5

3. *Proposition #1: Further Considerations*

   3a. Quadratic Considerations.. p. 6

   3b. The Lower Solution ……………………………………………………………………….p. 7

4. *Genetic Susceptibility in Women and Men – Variance Considerations .... p. 9*

5. *Genetic Susceptibility for the (H+) / (H−) Partition……..…......… p.12*

6. *Environmental Factors in MS*

   6a. Re-expressing Penetrance at Time-period #1 in terms of Time-period #2 .... p.14

   6b. Determining the Limiting Values for Penetrance……………………………............. p.14

   6c. Assessing the Environmental Threshold for MS in Men and Women.............................. p.15

   6d. Assessing the Increase in P(MS) for Canada (1945 – 1980)... p.16

   6e. Proportional vs. Non-proportional Hazard-rates for Men and Women......................… p.17

7. *Uniqueness of Susceptible Genotypes in the Population……….………........................…… p.18*

1. Penetrance Adjustment for the Shared Environment of *MZ*-Twins

Conclusions: 1. $P(MS \mid IG_{MS}) = \{P(MS \mid S_{MS}) / P(MS \mid DZ_{MS})\} * P(MS \mid MZ_{MS})$

Argument: We can break down a necessary and sufficient environmental exposure (*E*), into three components $(E_1, E_2, \text{ and } E_3)$. The component ($E_1$) is that part of this exposure shared exclusively by twins – i.e. the *IU* and certain (mostly early) post-natal environments; (E_2) is that part shared by the population generally; and (E_3), that part due to the shared familial micro-environment of siblings. Because twins are also siblings and, thus, share the same familial micro-environment, it will be the case that:

$$P(E_3 \mid MZ_{MS}) = P(E_3 \mid DZ_{MS}) = P(E_3 \mid S_{MS})$$

Notably, however, the familial micro-environment (E_3) seems to have little or no impact on the likelihood of developing MS [65-71]. In this circumstance, therefore:

$$P(E) = P(E_1, E_2, E_3) = P(E_1, E_2)$$

We also define $\{P(MS \mid IG_{MS})\}$ to be the *MZ*-twin concordance rate, adjusted to exclude the impact of their shared *IU* and post-natal environments (E_1). This relationship can be stated (*see Main Text*) as:

$$P(MS \mid MZ_{MS}) = P(MS, E_1, E_2 \mid MZ_{MS}) = P(E_1 \mid MZ_{MS}) * P(MS, E_2 \mid E_1, MZ_{MS})$$

where: $P(MS \mid IG_{MS}) = P(E_1) * P(MS, E_2 \mid E_1, MZ_{MS})$

Because both *MZ-twins* and *DZ-twins* share (E_1) – assuming that a sufficient (E_1) exposure is similar across susceptible individuals – and because both siblings and *DZ-twins* share the same genetic relationship with each other but don't share (E_1), we expect that:

$$P(E_1 \mid MZ_{MS}) = P(E_1 \mid DZ_{MS}); \quad P(E_1 \mid S_{MS}) = P(E_1); \quad \text{and: } P(MS, E_2 \mid E_1, DZ_{MS}) = P(MS, E_2 \mid E_1, S_{MS})$$

In this case: $P(MS \mid DZ_{MS}) = P(MS, E_1, E_2 \mid DZ_{MS}) = P(E_1 \mid MZ_{MS}) * P(MS, E_2 \mid E_1, DZ_{MS})$

and: $P(MS \mid S_{MS}) = P(MS, E_1, E_2 \mid S_{MS}) = P(E_1) * P(MS, E_2 \mid E_1, DZ_{MS})$

so that: $P(MS \mid S_{MS}) / P(MS \mid DZ_{MS}) = P(E_1) / P(E_1 \mid MZ_{MS})$

And therefore: $P(MS \mid IG_{MS}) = \{P(MS \mid S_{MS}) / P(MS \mid DZ_{MS})\} * P(MS \mid MZ_{MS})$

1.1. Penetrance Adjustments for different Population Partitions

Conclusions: 1. The penetrance-adjustment for the shared early environment of *MZ*-twins is similar for both (*Male/Female*) and (*H+/H−*) partitions.

Argument: Using the above relationship, from the Canadian population data set (*Table 3; Fig 3; Main Text*), we have already estimated the value of $\{P(MS \mid IG_{MS})\}$ as:

$$P(MS \mid IG_{MS}) = (2.9 / 5.4) * 0.25 = 0.25 / 1.86 = 0.134$$

In the more limited data from the *HLA* partition (*Table 3; Fig 3; Main Text*), this estimate becomes:

$$P(MS \mid IG_{MS}) = (2.9/5.4)*0.30 = 0.30/1.86 = 0.161$$

Although these adjustments provide an estimate for the impact of the *IU* and early post-natal environment considering the population as a whole, the same adjustment may not be appropriate for every partition of that population. Using the data in *Table 3; Fig 3* (*Main Text*), however, certain partition-specific adjusted penetrance values can be estimated and these are quite similar for both partitions (*see below*).

1.1a. Penetrance Adjustments for the (*H+/ H−*) Partition

Conclusions: $P(MS \mid H+, IG_{MS}) = P(MS \mid H+, MZ_{MS})/1.84$
$P(MS \mid H-, IG_{MS}) = P(MS \mid H-, MZ_{MS})/1.87$

Argument: Using the data in *Table 3; Fig 3* (*Main Text*), we can define two parameters $(e \geq 1)$ and $(f \geq 1)$ such that:

$$P(MS \mid H+, IG_{MS}) = P(MS \mid H+, MZ_{MS})/e$$
$$P(MS \mid H-, IG_{MS}) = P(MS \mid H-, MZ_{MS})/f$$

In this case we can deconstruct the term $\{P(MS, H+ \mid IG_{MS})\}$ in two different ways:

$$P(MS, H+ \mid IG_{MS}) = P(H+ \mid IG_{MS}) * P(MS \mid H+, IG_{MS}) = 0.43*0.31/e = 0.133/e$$

and: $$P(MS, H+ \mid IG_{MS}) = P(MS \mid IG_{MS}) * P(H+ \mid MS, IG_{MS}) = 0.161*0.45 = 0.072$$

Combining these two equations leads to: $e = 0.133/0.072 = 1.84$

Similarly: $$P(MS, H- \mid IG_{MS}) = P(H- \mid IG_{MS}) * P(MS \mid H-, IG_{MS}) = 0.57*0.29/f = 0.165/f$$

and: $$P(MS, H- \mid IG_{MS}) = P(MS \mid IG_{MS}) * P(H- \mid MS, IG_{MS}) = 0.161*0.55 = 0.089$$

leading to: $f = 0.165/0.089 = 1.87$

1.1b. Penetrance adjustments for the (*F/ M*) partition

Conclusions: $P(MS \mid F, IG_{MS}) = P(MS \mid F, MZ_{MS})/1.82$

$P(MS \mid M, IG_{MS}) = P(MS \mid M, MZ_{MS})/2.0$

Argument: Similar to the analysis (*above*), using the data in *Table 3;Fig 3* (*Main Text*), we can, again, define two parameters $(e \geq 1)$ and $(f \geq 1)$ such that:

$$P(MS \mid F, IG_{MS}) = P(MS \mid F, MZ_{MS})/e$$
$$P(MS \mid M, IG_{MS}) = P(MS \mid M, MZ_{MS})/f$$

Again, we can deconstruct the term $\{P(MS,F\,|\,IG_{MS})\}$ in two different ways:

$$P(MS,F\,|\,IG_{MS}) = P(F\,|\,IG_{MS}) * P(MS\,|\,F,IG_{MS}) = 0.66*0.34/e = 0.224/e$$

and: $$P(MS,F\,|\,IG_{MS}) = P(MS\,|\,IG_{MS}) * P(F\,|\,MS,IG_{MS}) = 0.134*0.92 = 0.123$$

Combining these two equations leads to: $e = 0.224/0.123 = 1.82$

Similarly: $$P(MS,M\,|\,IG_{MS}) = P(M\,|\,IG_{MS}) * P(MS\,|\,M,IG_{MS}) = 0.34*0.07/f = 0.024/f$$

and: $$P(MS,M\,|\,IG_{MS}) = P(MS\,|\,IG_{MS}) * P(M\,|\,MS,IG_{MS}) = 0.134*0.08 = 0.012$$

leading to: $f = 0.024/0.012 = 2.0$

2. Enrichment of Genotypes – Partitioning (*G*) into Subsets (*G1*) & (*G2*)

2a. The 1st Enrichment

Conclusions: $P(MS\,|\,G1) = P(MS\,|\,G2)$ if and only if: $P(G1\,|\,MS,G) = P(G1\,|\,G)$

$P(MS\,|\,G1) > P(MS\,|\,G2)$ if and only if: $P(G1\,|\,MS,G) > P(G1\,|\,G)$

. ***Argument:*** The subset (*G*) can be partitioned into two mutually exclusive subsets (*G1*) and (*G2*), such that: $P(G) = P(G1) + P(G2)$ or: $1 = P(G1\,|\,G) + P(G2\,|\,G)$

For the 1st enrichment stage, we can define two constants, (*a*) and (*b*) such that:

$$P(MS\,|\,G1) = a * P(MS\,|\,G) \quad \text{and:} \quad P(MS\,|\,G2) = b * P(MS\,|\,G)$$

where (*a*) and (*b*) are related such that:

$$P(MS\,|\,G) = P(MS,G1\,|\,G) + P(MS,G2\,|\,G)$$

or: $$1 = a * P(G1\,|\,G) + b * P(G2\,|\,G)$$

and, thus: $a = 1$ if and only if: $b = 1$

also: $a > 1$ if and only if: $b < 1$ (and *vice versa*)

and finally: $P(MS\,|\,G1)/P(MS\,|\,G2) = (a/b)$

Moreover, for any partition:

$$P(G1\,|\,MS,G) = P(G1,MS,G)/P(MS,G) = P(G1,G) * P(MS\,|\,G,G1)/P(MS,G)$$
$$= P(G1\,|\,G) * a * P(MS\,|\,G)/P(MS\,|\,G) = a * P(G1\,|\,G)$$

And similarly: $P(G2\,|\,MS,G) = b * P(G2\,|\,G)$

so that: $P(G1\,|\,MS,G)/P(G2\,|\,MS,G) = (a/b) * P(G1\,|\,G)/P(G2/G)$

Consequently, when: $a = b = 1$ then: $P(G1\,|\,MS,G) = P(G1\,|\,G)$ and: $P(G2\,|\,MS,G) = P(G2\,|\,G)$

However, if: $\sigma_X^2 > 0$; then there must be at least one partition, for which both (*G1*) and (*G2*) are non-empty and (*suitably defined*), for which: $(a > 1 > b)$, and therefore:

$$P(G1 \mid MS,G) > P(G1 \mid G) \quad \text{and:} \quad P(G2 \mid MS,G) < P(G2 \mid G)$$

Thus, any subset of more penetrant genotypes (*G1*) will be enriched in the (MS,G) subset relative to a less penetrant subset (*G2*). Also, equally penetrant subsets will not be enriched relative to each other. Clearly, the reciprocal arguments also hold so that:

$$P(G1 \mid MS,G) = P(G1 \mid G) \quad \text{if and only if:} \quad P(MS \mid G1) = P(MS \mid G2)$$

and: $$P(G1 \mid MS,G) > P(G1 \mid G) \quad \text{if and only if:} \quad P(MS \mid G1) > P(MS \mid G2)$$

2b. The 2nd Enrichment

Conclusions:

$$P(MS \mid G1, IG_{MS}) = P(MS \mid G2, IG_{MS}) \quad \text{if and only if:} \quad P(G1 \mid MS, IG_{MS}) = P(G1 \mid MS)$$

$$P(MS \mid G1, IG_{MS}) > P(MS \mid G2, IG_{MS}) \quad \text{if and only if:} \quad P(G1 \mid MS, IG_{MS}) > P(G1 \mid MS)$$

Argument: Similar to the 1st enrichment stage, we can define two constants, (*v*) and (*w*), such that: $$P(MS \mid G1, IG_{MS}) = v * P(MS \mid IG_{MS}) \quad \text{and:} \quad P(MS \mid G2, IG_{MS}) = w * P(MS \mid IG_{MS})$$

in which case: $$P(MS \mid IG_{MS}) = P(MS, G1 \mid IG_{MS}) + P(MS, G2 \mid IG_{MS})$$

or: $$1 = v * P(G1 \mid MS) + w * P(G2 \mid MS) = a * v * P(G1 \mid G) + b * w * P(G2 \mid G)$$

And, similar to the 1st enrichment stage, for the 2nd enrichment stage:

$$v = 1 \quad \text{if and only if:} \quad w = 1$$

and. also: $$v > 1 \quad \text{if and only if:} \quad w < 1 \qquad \text{(and } \textit{vice versa}\text{)}$$

Moreover: $$P(G1 \mid MS, IG_{MS}) = P(G1, MS, IG_{MS}) / P(MS, IG_{MS}) = v * P(G1 \mid MS, G) = v * a * P(G1 \mid G)$$

and: $$P(G2 \mid MS, IG_{MS}) = w * P(G2 \mid MS, G) = w * b * P(G2 \mid G)$$

so that: $$P(G1 \mid MS, IG_{MS}) / P(G2 \mid MS, IG_{MS}) = (v / w)(a / b)\{P(G1) / P(G2)\}$$

2c. Combining Enrichment Results

Conclusions: Defining the additional parameters $(g, p, r, s, \; \& \; t)$ then:

1. $t = (a / b)\{p / (1 - p)\} = (x_1 / x_2)\{p / (1 - p)\}$
2. $P(MS) / g = x = p(x_1) + (1 - p)(x_2)$
3. $(a / b) = (v / w)(s / r)$ where: $0.5 < (s / r) < 2$

Argument: For simplicity of notation, in addition to those parameters already defined, we

introduce five additional parameter abbreviations $(g, p, r, s \ \& \ t)$ – *see Table 2, Main Text* – such that:

$$g = P(G); \ \ p = P(G1 | G); \ \ r = (x_1')/(x_1); \ \ s = (x_2')/(x_2); \ \text{and:} \ \ t = P(G1 | MS)/P(G2 | MS)$$

From *#2a* (*above*): $t = P(G1 | MS)/P(G2 | MS) = (a/b) * P(G1 | G)/P(G2/G)$

or, equivalently: $t = (a/b)\{p/(1-p)\} = (x_1/x_2)\{p/(1-p)\}$ (*Equation #1*)

and also: $P(MS | G) = P(MS)/g = x = p(x_1) + (1-p)(x_2)$ (*Equation #2*)

If the expected penetrance of the ($G1$) and ($G2$) sub-subsets differ significantly from each other, then we are assuming that each, considered separately, conforms to the Upper Solution (*see Methods, Main Text*). If so, then from *Proposition #1* (*Main Text*):

$$1 \le r < 2 \ \text{ and, also: } \ 1 \le s < 2$$

Moreover: $(r/s) = \dfrac{(x_1')/(x_1)}{(x_2')/(x_2)} = (x_1'/x_2')(x_2/x_1) = (v/w)(b/a)$

so that: $(a/b) = (v/w)(s/r)$ where: $0.5 < (s/r) < 2$ (*Equation #3*)

3. Proposition #1: Further Considerations

3a. Quadratic Considerations

Conclusions: 1. $$x_1 = \frac{x + \sqrt{x^2 - \{1 + (r/s)(1-p)/p\}\{(x^2 - xx'(1-p)/s\}}}{p + (r/s)(1-p)}$$

2. $$x_2 = \frac{x - \sqrt{x^2 - \{1 + (s/r)p/(1-p)\}\{(x^2 - xx'p/r\}}}{(1-p) + (s/r)p}$$

Argument: Restating *Equation #2* (*see #2c above*):

$$x = p(x_1) + (1-p)(x_2)$$

so that: $x_2 = [x - p(x_1)]/(1-p)$ (*Equation #4a*)

In addition: $x' = P(MS | G, IG_{MS}) = P(MS, G1 | G, IG_{MS}) + P(MS, G2 | G, IG_{MS})$

therefore: $P(MS, G1 | G, IG_{MS}) = P(G1 | G, IG_{MS}) * (x_1') = P(G1 | G, MS) * (x_1')$

where: $P(G1 | G, MS) = P(G1, MS | G)/P(MS | G) = p(x_1)/x$

similarly: $P(G2 | G, MS) = (1-p)(x_2)/x$

so that: $xx' = p(x_1)(x_1') + (1-p)(x_2)(x_2') = pr(x_1)^2 + (1-p)s(x_2)^2$

or: $(x_2)^2 = [xx' - pr(x_1)^2]/(1-p)s$ (*Equation #4b*)

Consequently, we have two different estimates for $(x_2)^2$ – i.e., *Equations #4a and #4b, above*.

Combining these two estimates yields:

$$[\{x - p(x_1)\}/(1-p)]^2 = (x_2)^2 = \{xx' - pr(x_1)^2\}/(1-p)s$$

or: $$[\{x - p(x_1)\}]^2 = \{xx' - pr(x_1)^2\}(1-p)/s = \{xx'(1-p)/s\} - \{(r/s)p(1-p)(x_1)^2\}$$

and, finally: $$x^2 - 2xp(x_1) + p^2(x_1)^2 - xx'(1-p)/s + (r/s)p(1-p)(x_1)^2 = 0$$

Rearrangement, yields a quadratic equation in (x_1) such that:

$$\{p^2 + (r/s)p(1-p)\}(x_1)^2 - \{2xp\}(x_1) + \{x^2 - xx'(1-p)/s\} = 0$$

Because of the constraint that: $(x_1 > x > x_2)$ – *see Methods, Main Text* – this is solved for (x_1) as:

$$x_1 = \frac{x + \sqrt{x^2 - \{1 + (r/s)(1-p)/p\}\{(x^2 - xx'(1-p)/s\}}}{p + (r/s)(1-p)} \qquad (\textit{Equation \#5a})$$

Equation #4a (*above*) can then be solved for (x_2). Alternatively, reframing the above argument, yields:

$$x_2 = \frac{x - \sqrt{x^2 - \{1 + (s/r)p/(1-p)\}\{(x^2 - xx'p/r\}}}{(1-p) + (s/r)p} \qquad (\textit{Equation \#5b})$$

3b. The Lower Solution.

Conclusions: $\forall\{x < x'/2\}:\ p > (2 - b^2 s)/(a^2 r - b^2 s)$

$\forall\{x > x'/2\}:\ p < (2 - b^2 s)/(a^2 r - b^2 s)$

$\forall\{x < x'/2\}:\ 0.025 \le P(G) \le 0.18$

and: $\forall\{x < x'/2\}:\ 0.006 \le P(G1) \le 0.063$

Argument: The values for $\{x', x_1', x_2',\ P(MS)\ \&\ P(F \mid MS)\}$ are based upon observation and, as such, subject to error. To simplify our notation, we use the parameter abbreviations in *Table 2* (*Main Text*); noting, for clarity, that: $p = P(G1 \mid G)$; $a = x_1/x$; $b = x_2/x$; $r = x_1'/x_1$; and: $s = x_2'/x_2$

In this case: $P(G1 \mid IG_{MS}) = P(G1 \mid MS) = P(G1) * P(MS \mid G1)/P(MS) = px_1/x = pa$

also: $P(MS \mid G1, IG_{MS}) = x_1' = (x_1/x)(x_1'/x_1)(x) = arx$

Similarly: $P(G2 \mid IG_{MS}) = (1-p)b$ and: $P(MS \mid G1, IG_{MS}) = x_2' = bsx$

Also: $x' = P(G1 \mid IG_{MS}) * arx + P(G2 \mid IG_{MS}) * bsx = \{pa^2 r + (1-p)b^2 s\} * x$

Because from *Proposition #1* (*Main Text*), the Lower Solution (where: $x'/x > 2$) requires that:

$$x'/x = pa^2 r + (1-p)b^2 s > 2 \quad \text{or:} \quad p > (2 - b^2 s)/(a^2 r - b^2 s)$$

Similarly, the Upper Solution (where: $x'/x < 2$) requires that: $p < (2 - b^2 s)/(a^2 r - b^2 s)$

Moreover, because these quadratic solutions (*Equations #5a & #5b, above*) depend upon the

values of four unknown variables $(r,\ s, p\ \&\ g)$ they cannot be solved uniquely. Nevertheless, a range of possible parameter values can be explored iteratively, using parameter combinations that cover (for each unknown parameter) their entire plausible ranges, taking into account possible errors in the observations, and incorporating certain constraints on possible solutions.

For example, in this analysis (*similar to #4, below*), we used the observational data on the gender partition (*Table 3; Fig 3, Main Text*), assigning women to the $(G1)$ subset {i.e., $(G1) = P(F,G)$ }. In addition, the parameters $\{g,\ p,\ P(MS),\ \&\ P(F \mid MS)\}$, were assigned the plausible ranges of: $(0.0001 \le g = P(G) \le 1)$; $(0.0001 \le p = P(F \mid G) \le 1)$, $(0.002 \le P(MS) \le 0.006)$, and $(0.60 \le P(F \mid MS) \le 0.80)$ respectively. Also, we considered the observed values of (x'), (x_1'), and (x_2') acceptible if each was within (±25%) of their observed values (i.e., $0.107 \le x' \le 0.161$; $0.134 \le x_1' \le 0.223$; and $0.028 \le x_2' \le 0.042$ repectively) – *see Table 3; Fig 3, Main Text* . And, finally, we assumed that sub-subsets (F,G) and (M,G), considered separately, each has a distribution of penetrance values, which conforms to the Upper Solution (*see Methods, Main Text*). This assumption restricts the possible ranges for the (r) and (s) parameters to $(1 \le r < 2)$ and $(1 \le s < 2)$ respectively (*see Proposition* #1; *Main Text*).

We then iteratively assigned, to each input parameter $\{g, p, x', r, s,\ \&\ P(MS)\}$, values which spaned each of the above ranges, solved *Equations #5a & #5b* (*above*) for each parameter combination, and determined which combinations satisfied the above constraints. From this analysis we conclude that:

$$\forall\{x < x'/2\}:\ \ 0.025 \le P(G) \le 0.18$$

and: $$\forall\{x < x'/2\}:\ \ 0.006 \le P(G1) \le 0.063$$

Thus, although Lower Solutions exist for which, $\{P(G) = 1\}$, none of these solutions match the constraints (*above*) placed by the observed the values of $\{x',\ x_1',\ x_2'\ \ \&\ P(F \mid MS)\}$ for the partition based on gender (*see #5, Main Text*). Indeed, this analysis demonstrated that:

$$\forall\{P(G) = 1\}:\ x_2' < 0.009$$

which is far removed from the actual observational data (*Table 3; Fig 3, Main Text*). Thus, the circumstance of $\{P(G) = 1\}$ seems to be excluded, even for Lower Solutions, except for the most extreme distributional circmstances. In earlier iterations of this analysis [3,4,51,52], we defined the (G) subset differently – i.e., $\forall G_i \in G:\ P(MS \mid G_i) \ge P(MS)$. We note that, for Lower Solutions in the present analysis, our older definition effectively corresponds to defining only members of the ($G1$)-subset as being "genetically susceptible" to MS.

4. Genetic Susceptibility in Women and Men – Variance Considerations

Conclusions: 1. The set {*X*} has, at least, a bimodal distribution

2. $0.145 \le P(MS \mid F,G) \le 0.187$
3. $0.017 \le P(MS \mid M,G) \le 0.034$
4. $0.18 \le P(F \mid G) \le 0.31$
5. $4.3 \le P(MS \mid F,G) / P(MS \mid F,G) \le 8.7$
6. $0.041 \le P(G) < 0.073$

Argument: Following the notation in *Table #2* (*Main Text*) and logic of *#2 & #3 (above)* and of *Proposition #1* (*Main Text*), we can again define a partition of the (*G*) subset into susceptible women $\{(G1)=(F,G)\}$ and susceptible men $\{(G2)=(M,G)\}$. The set {*X*} of penetrance values for members of the (*G*) subset (*Proposition #1* ; *Main Text*) is, at least, bimodal. Thus, from the observational data in *Table 3; Fig 3* (*Main Text*):

$$P(MS \mid F, MZ_{MS}) = 0.34 >> 0.067 = P(MS \mid M, MZ_{MS})$$

$$\chi^2 = 8.5 \;\; ; \;\; p = 0.0035$$

For this analysis, we assume that sub-subsets (*G*1) and (*G*2) meet conditions for the Upper Solution (*see Methods; Main Text*). From *Proposition #1 (Main Text)*, considering the (*M / F*) partition, using the estimated adjustments for the similar early environment of twins for these gender subgroups (*see #1.2b above*), and incorporating the data provided in *Table 3; Fig 3* (*Main Text*), it follows that:

$$0.093 < x_1 \le 0.187 \qquad (Equation\ \#6a)$$

and: $$0.017 < x_2 \le 0.034 \qquad (Equation\ \#6b)$$

These possible ranges for men and women don't overlap. Therefore, we have correctly defined the sub-subsets (*G1*) and (*G2*) *see above* – because, for this partition: $(x_1 > x > x_2)$ – *see Methods, Main Text*. The proportion of MS patients who are women from *Table 3; Fig 3* (*Main Text*) is 66%. For the WTCCC data this number is 72%. From the study of Orton and colleagues [56] out of Canada, in the most recent epoch, the percentage of MS patients who are women is 76%. From a recent prevalence estimate for the United States [44], the percentage of women among MS patients is 74%. Using the data from *Table 3; Fig 3* (*Main Text*), together with the above ranges for men and women, and from the definition of the (*G*) subset, we can estimate that:

$$P(MS,G \mid F) = P(MS \mid F) = \{P(F \mid MS) * P(MS)\} / P(F) \approx \{0.66 * 0.003\} / 0.5 = 0.004$$

Because: $P(G \mid F) = P(MS,G \mid F) / P(MS \mid F,G)$

Therefore: $0.021 = 0.004 / 0.187 \le P(G \mid F) < 0.004 / 0.093 = 0.043$

And similarly: $0.060 = 0.002 / 0.034 \le P(G \mid M) < 0.002 / 0.017 = 0.118$

From this, the upper limit for the quantity $\{P(F \mid G)\}$ can be estimated from *Table 3; Fig 3* (*Main Text*):

Such that: $P(G \mid F)/P(G \mid M) = P(F \mid G)/P(M \mid G) = p/(1-p) < 0.043/0.060 = 0.717$

where: $1 + P(F \mid G)/P(M \mid G) = 1/P(M \mid G) = 1/(1-p) < 1 + 0.717 = 1.717$

so that: $p = P(F \mid G) < 0.717/1.717 = 0.42$

However, there are four serious concerns about undertaking any calculations that use the limits for $(x_1$ and: $x_2)$ set forth by *Equations #6a & #6b*. First, in the making above calculation, we are positing and an extreme and tri-modal distribution for the set $\{X\}$ – i.e., not the unimodal or bimodal distributions under primary consideration in this manuscript. Thus, this calculation, envisions a distribution, in which half of the women have a penetrance of slightly greater than zero and the other half have a uniform penetrance of (x_1') – i.e. women have the maximum variance possible – and in which each of the men has exactly the same penetrance of (x_2'), which is intermediate between these two extremes – i.e. men have a zero variance.

Second, such an extreme distribution seems very unlikely, especially for circumstances in which partitioning the (G) *subset* by a different MS-associated characteristic – i.e., *HLA*-status (*see #5, below*) – doesn't even give a hint of the bimodal nature of $\{X\}$.

Third, it is not possible that the variance of penetrance values for the (F,G) subset to be at its maximum value. Thus, because $(x' < x_1')$, the maximum variance for the $(G1)$ subset – $(x_1'/2)^2$ – exceeds the maximum total variance possible for the entire (G) *subset* – $(x'/2)^2$. Consequently, the lower limit for the value of (x_1) in *Equation #6a* – i.e., at its maximum possible variance – must be too low.

And fourth, some of the maximum possible variance in the $\{X\}$ set must be accounted for by the separation of (x_1) from (x_2). This will also increase the lower limit of (x_1). To see this, suppose that the $\{X\}$ set is bimodal. And further suppose that, of the (m) members of the (G) subset, $(ii = 1,2,3,...,g_1)$ belong to the $(G1)$ subset and $(jj = 1,2,3,...,g_2)$ belong the mutually exclusive $(G2)$ subset. In this case, $(p = g_1/m)$ and the variance of $\{X\}$ – *see Proposition #1; Main Text* – is:

$$\sigma_X^2 = E(x_i - x)^2 = (1/m)\sum_{i=1}^{m}(x_i - x)^2 = (1/m)\sum_{ii=1}^{g1}(x_{ii} - x)^2 + (1/m)\sum_{jj=1}^{g2}(x_{jj} - x)^2$$

For the $(G1)$ subset, following a standard development of variance relationships [57], this becomes:

$$(1/m)\sum_{ii=1}^{g1}(x_{ii} - x)^2 = (1/m)\sum_{ii=1}^{g1}[(x_{ii} - x_1) + (x_1 - x)]^2 = (1/m)\sum_{ii=1}^{g1}(x_{ii} - x_1)^2 + (1/m)\sum_{ii=1}^{g1}(x_1 - x)^2$$

or: $$(1/m)\sum_{ii=1}^{g1}(x_{ii} - x)^2 = (g_1/m)\sigma_{x1}^2 + (g_1/m)(x_1 - x)^2 = p\sigma_{x1}^2 + p(x_1 - x)^2$$

Similarly: $$(1/m)\sum_{jj=1}^{g2}(x_{jj}-x)^2=(1-p)\sigma_{x2}^2+(1-p)(x_2-x)^2$$

Consequently: $$\sigma_X^2=\{p\sigma_{x1}^2+(1-p)\sigma_{x2}^2\}+\{p(x_1-x)^2+(1-p)(x_2-x)^2\}$$

Thus, part of the set {*X*} variance is accounted for by the term: $\{p(x_1-x)^2+(1-p)(x_2-x)^2\}$. This will also cause the lower limit for the value of (x_1) to be higher than expressed in that *Equation #6a*. For example, we can define the residual variance (R_v) as:

$$R_v=\sigma_X^2-p(x_1-x)^2-(1-p)(x_2-x)^2 \qquad \textit{Equation \#6}$$

where: $\sigma_X^2=x(x'-x)$; $\sigma_{x1}^2=x_1(x_1'-x_1)$; $\sigma_{x2}^2=x_2(x_2'-x_2)$; $x=px_1+(1-p)x_2$

As before, the upper limit for $P(G1|G)=P(F|G)$ can be established using the maximum value for (x_2) – i.e., where: $\sigma_{x2}^2=0$ – and the minimum value for (x_1) – i.e., where (σ_{x1}^2) accounts for all of the residual varaince (R_v). Under these circumstances, together with this definitions and these relationships, we have two equations in only two unknown (i.e., unobserved) parameters $(p$ and: $x_1)$, which can be solved uniquely. Thus, using (for the gender partition) the previously-defined relationship that:

$$t=P(F|MS)/P(M|MS)=0.66/0.34=1.94$$

and using the Upper Solution (*Proposition #1, Main Text*), at the boundry, $(x_1$ and: $p)$ become:

$$p=t/\{(x_1/x_2)+t\} \qquad \textit{Equation \#1; re-expressed}$$

and: $$x_1=\frac{(x_1')+\sqrt{(x_1')^2-4R_v}}{2}$$ where: $\sigma_{x1}^2\le R_v$

In turn, these two equations can be solved iteratively by inserting our initial lower-limit estimate of $(x_1=0.093)$ and the estimate of $(x_2=x_2')$ when $(\sigma_{x2}^2=0)$ into the 1st equation and then estimating a new lower limit for (x_1) from the 2nd equation and *Equation #6* , taking into account the condition that $(\sigma_{x1}^2\le R_v)$. This new lower-limit estimate for (x_1) can then be re-inserted into the 1st equation and the process repeated until the two estimates for (x_1) converge and are identical. Initially assuming that the entire residual variance (R_v) is confined to penetrance values in the ($G1$)-subset (i.e., $\sigma_{x2}^2=0$), and using *Equation #6* to determine the magnitude of (R_v), this process converges on the solution that:

$x_1=0.145$; $p=0.31$; and: $(a/b)=4.3$

Permitting the variance (σ_{x2}^2) to increase to its maximum possible value of: $(x_2'/2)^2$, with

(σ^2_{x1}) accounting for all of the remaining R_V, only alters these conclusions for $\{p$ and: $(a/b)\}$ such that:

$$x_1 = 0.146 \ ; \quad p = 0.18 \ ; \text{ and: } (a/b) = 8.7$$

Consequently: $0.145 \le x_1 \le 0.187$; $0.017 \le x_2 \le 0.034$; $0.18 \le p \le 0.31$; and: $4.3 \le (a/b) \le 8.7$

And, from this we conclude that:

$$0.011 = 0.002/0.187 \le P(G,F) < 0.002/0.145 = 0.014$$

and: $$0.030 = 0.001/0.034 \le P(G,M) < 0.001/0.017 = 0.059$$

so that: $$0.041 \le P(G) = P(G,F) + P(G,M) < 0.073$$

Notably, these limits are derived by including all solutions (both Upper and Lower), in which (*G1*) and (*G2*) have significantly different penetrances and where each follows an Upper Solution. However, if the distribution of $\{X\}$ follows an Upper Solution, those limits will still apply (*see Main Text*), although the slightly different estimates for $P(G)$ would need to be reconciled. Two possibilities suggest themselves. First, for Lower Solution distributions, these limiting solutions for $P(G)$ describe non-unimodal distributions for both ($G1$) and $G2$). If we were to consider only the possibility that the distribution of penetrance values in ($G1$) and $G2$) each meet the conditions for a unimodal distribution (*see Proposition #1, Main Text*) then the limits provided by these equations would be:

$$0.163 \le x_1 \le 0.187 \ ; \ 0.029 \le x_2 \le 0.034 \ ; \ 0.23 \le p \le 0.28 \ ; \text{ and: } 4.8 \le (a/b) \le 6.4$$

And, in this case, the limits for $P(G)$ would be: $0.041 \le P(G) < 0.047$

And second, the estimate from *Table 3; Fig 3* (*Main Text*) for the quantity $\{P(MS \mid M, IG_{MS})\}$, is based on only two concordant twins, and, thus, this estimate seems likely to be the least reliable of any in the *Table.* If this estimated penetrance were doubled, there would still be an excess of men in (*G)*

such that: $$0.31 \le P(F \mid G) \le 0.47$$

but also: $$0.026 \le P(G) \le 0.043$$

5. Genetic Susceptibility for the (*H*+) / (*H*−) Partition

Conclusion: $P(G \mid H+) \approx 3.35 * P(G \mid H-)$

Argument: From the WTCCC it is apparent that there is considerable enrichment of (*H*+) status during the 1st enrichment stage when moving from the general population to an MS population.

Thus: $$P(MS,G \mid H+) / P(MS,G \mid H-) = 3.35$$

This relationship can be expressed alternatively as:

$$\{P(G \mid H+) / P(G \mid H-)\} * \{P(MS \mid G,H+) / P(MS \mid G,H-)\} = 3.35$$

What this re-expression makes it clear that the enrichment of (H+)-status in MS can occur in one, or both, of two possible ways. First, (H+) membership could make membership in the (G) subset more likely than it is for the ($H-$) subset – i.e., it is due to an impact on the ratio of: $P(G \mid H+) / P(G \mid H-)$. Second, members of the $(G, H+)$ subset may have a greater penetrance for MS than members of the $(G, H-)$ subset – i.e., it is due to an impact on the ratio of: $P(MS \mid G, H+) / P(MS \mid G, H-)$.

As discussed in *#4* (*above*), the set {X}, based on the partition of the (G) subset by gender, is at least bimodal. Therefore, there is a continuing enrichment of women during both the 1st and the 2nd stage of enrichment as demonstrated (*see Table 3; Fig 3; Main Text*) by the following relationships:

$$P(F \mid MS) / P(F) = 1.32 \approx 1.39 = P(F \mid MS, IG_{MS}) / P(F \mid MS)$$

By contrast, the enrichment of (H+)-status disappears during the (observable) 2nd stage such that:

$$P(H+ \mid MS) / P(H+) = 1.79 >> 1.05 = P(H+ \mid MS, IG_{MS}) / P(H+ \mid MS)$$

However, if either: $P(F, H+ \mid G) > P(F, H- \mid G)$ or: $P(F, H- \mid G) > P(F, H+ \mid G)$

then the group with the greater proportion of women will be enriched (due to the considerably greater penetrance in women – *see #4, above*) during the 2nd enrichment stage. This should be true even if (H+) has little impact by itself. The enrichment should be even greater if (H+) has an additional impact although there is little observational evidence for this. Thus, based on the data of *Table 3; Fig 3* (*Main Text*):

$$P(F, H+ \mid MS) = 0.34 \approx 0.35 = P(F, H- \mid MS)$$

and: $$P(MS \mid F, H+, IG_{MS}) = 0.21 \approx 0.24 = P(MS \mid F, H-, IG_{MS})$$

Taken together, these two observations suggest that there is little, or no, enrichment of (H+) status during the 2nd stage in women. Consequently, gender-status must be approximately balanced within the two *HLA*-subgroups such that:

$$P(F, H+, G) \approx P(F, H-, G)$$

In which case: $P(G \mid F, H+) * P(F, H+) \approx P(G \mid F, H-) * P(F, H-)$

Using the WTCCC control data, this relationship translates to:

$$P(G \mid F, H+) * 0.23 \approx P(G \mid F, H-) * 0.77$$

or: $$P(G \mid F, H+) \approx 3.35 * P(G \mid F, H-)$$

Similarly, in WTCCC controls: $P(H+ \mid F) \approx P(H+ \mid M)$, so that for men also:

$$P(G \mid M, H+) \approx 3.35 * P(G \mid M, H-)$$

Therefore: $$P(G \mid H+) \approx 3.35 * P(G \mid H-)$$

Consequently, the large majority of the enrichment of (H+) status in MS seems to be due to the fact that (H+)-membership makes membership in the (G) subset more likely than it is for (H−) membership. By contrast, (H+)-status seems to have very little impact on penetrance.

6. Environmental Considerations in MS-Pathogenesis for Men and Women

6a. Re-expressing Penetrance at *Time-period #1* in terms of *Time-period #2*

Conclusions: 1. $Zw_1 = P(MS,E\,|\,G,F)_1 = \{P(F\,|\,MS)_1 / P(F\,|\,MS)_2\} * C * (Zw_2)$

2. $Zm_1 = P(MS,E\,|\,G,M)_1 = \{P(M\,|\,MS)_1 / P(M\,|\,MS)_2\} * C * (Zm_2)$

Argument: From the definitions (*Main Text*) of (Zw_1), (Zw_2) and of the subsets (G) and (E):

$$P(MS,E,G,F) = P(MS,F)$$

and, thus: $Zw_2 = P(MS,E\,|\,G,F)_2 = P(MS,F)_2 / P(G,F) = P(MS)_2 * P(F\,|\,MS)_2 / P(G,F)$

Because, by the definition of (C) – see *Main Text*: $P(MS)_1 = C * P(MS)_2$

Therefore: $Zw_1 = P(MS,E\,|\,G,F)_1 = C * P(MS)_2 * P(F\,|\,MS)_1 / P(G,F)$

And, thus: $Zw_1 = (Zw_2) * C * \{P(F\,|\,MS)_1 / P(F\,|\,MS)_2)\}$

Similarly: $Zm_1 = (Zm_2) * C * \{P(M\,|\,MS)_1 / P(M\,|\,MS)_2)\}$

6b. Determining the Limiting Values for Penetrance

Conclusions: 1. $d = (Zw_2) * \{1 - [P(F\,|\,MS)_1 / P(F\,|\,MS)_2] * C * e^{-1}\} / (1 - e^{-1})$

2. $c = (Zm_2) * \{1 - [P(M\,|\,MS)_1 / P(M\,|\,MS)_2] * C * e^{-1}\} / (1 - e^{-1})$

Argument: From the *Main Text*, using the scale ($a^{app} = R*a$) for *women*:

$$Zw_2 = P(MS,E\,|\,G,F)_2 = d * \{1 - e^{-(a1^{app}+1-\lambda w)}\}$$
$$Zw_1 = P(MS,E\,|\,G,F)_1 = d * \{1 - e^{-(a1^{app}-\lambda w)}\}$$

These two equations can be re-arranged to yield:

$$(Zw_2 - d) / d = -\{e^{-(a1^{app}-\lambda w)}\} * e^{-1}$$
$$(Zw_1 - d) / d = -\{e^{-(a1^{app}-\lambda w)}\}$$

Dividing these two equations yields: $(Zw_2 - d) / (Zw_1 - d) = e^{-1}$

and with re-arrangement, this equation yields:

$$d = P(MS\,|\,G,E,F) = \{Zw_2 - Zw_1 * e^{-1}\} / (1 - e^{-1})$$

Substituting into this last equation for (Zw_1) from (*#6a*; *above*) yields:

$$d = (Zw_2) * \{1 - [P(F \mid MS)_1 / P(F \mid MS)_2] * C * e^{-1}\} / (1 - e^{-1})$$

Analogously: $$c = P(MS \mid G, E, M) = \{Zm_2 - Zm_1 * e^{-1}\} / (1 - e^{-1})$$

and: $$c = (Zm_2) * \{1 - [P(M \mid MS)_1 / P(M \mid MS)_2] * C * e^{-1}\} / (1 - e^{-1})$$

6c. Assessing the Environmental Threshold for MS in Men and Women

Conclusions: 1. $\lambda = \ln\{[1 - Zw_2 / d)] / [(1 - Zm_2 / c)]\}$

2. $\forall C > 0.50:\ 0.37 < \lambda < 4.67$; and, in fact: $\forall C > 0 : \lambda > 0$

Argument: Because the scales for the response-curves for women and men are initially assumed to be proportional they can be plotted on the same graph (*see Fig. 4; Main Text; see also #7 below*) and, when this is done on the (*a*) scale, the threshold (*x-intercept*) for men occurs at $\{(a, Zm) = (\lambda m, 0)\}$ and for women at $\{(a, Zw) = (\lambda w, 0)\}$. By the definitions of (*E*) and (*a*), one of these thresholds must occur at $\{(a, Z) = (0,0)\}$ – provided this exposure level is possible (*see Fig 4 & #7; Main Text*). However, these thresholds need not be the same and, therefore, we define the difference in threshold between women and men as: $(\lambda = \lambda w - \lambda m)$ such that, if women have a higher threshold than men: $(\lambda > 0)$. However, as noted (*above*), the (a) scale (for men) may be different than the (a^{app}) scale for women so that in order to plot them on the same graph requires the conversion of (a^{app}) units into (a) units. Because the *y-axis* is identical for both men and women, this conversion, depending upon the value of *R*, will cause the response curve for women to be stretched or compressed along the *x-axis* when converted into (*a*) units (without affecting the *y-axis* values), compared to their response curve when: $R = 1$. For women, on the (a^{app}) scale, the exponential curve passes through the points $(0,0)$, (a_1^{app}, Zw_1), (a_2^{app}, Zw_2), and (∞, d). Because the units on the *y-axis* are unchanged, this transformation into (*a*) units is defined by:

$$\forall a^{app} > 0 \ \&\ R > 0:\ (a^{app}, Zw) \rightarrow ([a^{app} / R], Zw) = (a, Zw)$$
$$\forall a^{app} = 0 \ \&\ R > 0:\ (a^{app}, Zw) = (0,0) \rightarrow (\lambda w, 0)$$

and: $$\forall a^{app} < 0:\ (a^{app}, Zw) \rightarrow (a, 0)$$

Thus, after conversion, the curve for women will pass through the points $(\lambda w, 0)$, (a_1, Zw_1), (a_2, Zw_2), and (∞, d). Because any two points define an exponential curve uniquely, there is only one curve that matches these conditions. *{NB: values of (Zw) are taken from the (a^{app}) scale so that, when plotted together with men on the (a) scale, depending upon the value of R, the curve will lie above or below that found when the scales are the same (i.e., R=1) – see Fig 4, Main Text }*. Moreover, by the

above transformation, (λw) is independent of R so that the condition $(R=1)$ can be used to estimate (λw) and, thus, responses at the second time-point can be expressed as:

$$Zw_2 = P(MS,E\,|\,G,F)_2 = d*\{1-e^{-(a1+1-\lambda w)}\} = d*\{1-e^{-(a1+1-\lambda-\lambda m)}\} \qquad (women)$$
$$Zm_2 = P(MS,E\,|\,G,M)_2 = c*\{1-e^{-(a1+1-\lambda m)}\} \qquad (men)$$

With re-arrangement, these equations become:

$$(Zw_2-d)/d = -e^{-(a1+1-\lambda m)}\}*e^{\lambda}$$
$$(Zm_2-c)/c = -e^{-(a1+1-\lambda m)}\}$$

Dividing these two equations yields:

$$(c/d)*[Zw_2-d)/(Zm_2-c)] = e^{\lambda}$$

So that: $\lambda = \ln\{(c/d)*[Zw_2-d)/(Zm_2-c)]\} = \ln\{[1-Zw_2/d)]/[(1-Zm_2/c)]\}$

For notational simplicity, we can define two expressions – (K_M) and K_F) – such that:

$$K_M = [P(M\,|\,MS)_1/P(M\,|\,MS)_2]*C*e^{-1}$$

and: $$K_F = [P(F\,|\,MS)_1/P(F\,|\,MS)_2]*C*e^{-1}$$

Substituting these into the using equations for (c) and (d) in *#6b* (*above*) and restating yields:

$$c = Zm_2*(1-K_M)/(1-e^{-1})$$

and: $$d = Zw_2*(1-K_F)/(1-e^{-1})$$

so that: $$(1-Zw_2/d)/(1-Zm_2/c) = [1-\{(1-e^{-1})/(1-K_F)\}]/[1-\{(1-e^{-1})/(1-K_M)\}]$$

Based on observational data [56]: $K_M > K_F$ so that: $1/(1-K_M) > 1/(1-K_F)$

and, thus: $[1-\{(1-e^{-1})/(1-K_F)\}] > [1-\{(1-e^{-1})/(1-K_M)\}]$

The value of (λ) depends only upon the value of (C) and the sex-ratio change over time so that, using the *above* equations for (λ) and applying *#6d* (*below*), we conclude that both:

$$\forall C > 0.50:\ 0.37 < \lambda < 4.67\ ;\ \text{and:}\ \forall C > 0: \lambda > 0$$

6d. Assessing the increase in MS-prevalence for Canada (1945 – 1980)

Conclusions: 1. $C = P(MS)_1/P(MS)_2 < 0.76$

2. $P(MS)_2 > (1/C)*P(MS)_1 > 1.32*P(MS)_1$

Argument: From *#6a* and *#6b* (*above*):

$$Zm_1 = (Zm_2)*C*P(M\,|\,MS)_1/P(M\,|\,MS)_2\}$$

and: $$c = P(MS\,|\,G,E,M) = \{Zm_2 - Zm_1*e^{-1}\}/(1-e^{-1})$$

Substituting the 1st equation into the 2nd, and noting that $\{Zm_2 < c\}$, yields:

$$Zm_2 < \{Zm_2 - [P(M \mid MS)_1 / P(M \mid MS)_2] * C * (Zm_2) * e^{-1}\} / (1 - e^{-1})$$

Dividing through by (Zm_2) and with re-arrangement this yields:

$$C < P(M \mid MS)_2 / P(M \mid MS)_1 = 0.238 / 0.313 = 0.76$$

Therefore: $$P(MS)_2 = (1/C) * P(MS)_1 = 1.32 * P(MS)_1$$

6e. Proportional vs. Non-proportional Hazard-rates for Men and Women

Conclusions:
1. The limiting-values of (***c***) and (***d***) are the same in either case
2. Non-proportional hazards require separate graphs for men and women
3. Non-proportional hazards imply different environmental factors
4. Proportional hazards imply similar environmental factors

Argument: From the separate definitions for the response curves in woman and men (*described above in #6b*), it follows directly that the limiting-values (***c***) and (***d***) depend only upon the independent variables (a) and (a^{app}) respectively. However, non-proportionality suggests that there is no known relationship between these two variables and, therefore, that no further comparative information can be gleaned. The response curves can be graphed separately but, because the relationship between the scales of the two is not known, they can't be placed on the same graph. Nevertheless, if the relationship between these two scales could be defined in some other manner, it might be possible to develop comparative information. In addition, non-proportionality would suggest that the environmental factors, which contribute to $P(E)$, are different for men and women.

By contrast, the assumption of proportional hazard rates directly implies that women have a higher environmental threshold than men *(Fig. 4; Main Text & see #6c above*) and such a circumstance suggests that men and women are responding to the same environmental events. Otherwise, if the necessary environmental factors were different for men and women, there must be some specific environmental conditions that favor MS-development in women over men and, in such a case, there could be no consistent difference in threshold. Rather, the existence of a threshold difference between men and women suggests that any gender-specific differences in MS-development depend only upon the degree (not the kind) of exposure. For example, perhaps, susceptible men develop MS with a lesser degree of vitamin D deficiency or with EBV infection occurring over a broader age-range compared to susceptible women.

Alternatively, there may be an environment-gender interaction such that susceptible men, in any given environment (i.e., E_T), are more likely to experience a sufficient exposure than susceptible women. For example, perhaps men are more likely to engage in "risky" behaviors compared to women, or that they are more likely to be "sun-averse" than women. Having said this, however, it is not clear how (or whether) "individual" differences in behavior (even if they are biologically driven) could lead to a

“population-level” difference in threshold (*Fig 4; Main Text*). More likely, any such interactions would have to be related to physiological differences between the genders.

Another possibility is that a small proportion of both susceptible men and women have “purely genetic” MS and that “environmental” MS begins for both genders at (0,0) – *see Main Text & Fig 4*.

7. Uniqueness of Susceptible Genotypes in the Population

An early GWAS of the WTCCC data identified a set of 102 non-*MHC* SNPs, for which one of the two SNP-variants at each location was significantly (and reliably) associated with MS [13]. Thus, including the “risk” haplotype (*H*+) in the *HLA* Class II region of the *MHC*, 103 “risk” locations were identified [13]. Among control subjects, there were, on average, 31 of these locations at which subjects were homozygous for the “non-risk” *SNP* variant, 32 locations at which subjects were homozygous for the “risk” *SNP*-variant, and 40 locations at which control subjects were heterozygous. By contrast, among cases, these numbers were 29, 34, and 40 (respectively). Even if one considers individuals who are either homozygous or heterozygous for the “risk” *SNP*-variant at all locations to have the same genotype, there are still a huge number of possible combinations. Thus, in this circumstance there are:

$$\left(\frac{103}{74}\right) = 3.4 * 10^{25}$$

possible combinations. Nevertheless, it is clear, first, that heterozygotes and homozygotes carry different “risks” for some locations (e.g., *H*+), second, that many of the associated loci have multiple alleles (e.g., the *MHC*), and finally, that more than 200 genetic loci are now known to be MS-associated [5-14,24]. Each of these facts will hugely increase the number of possible genetic combinations of the MS-associated loci.

With fewer than $\{10^{10}\}$ people in the entire world, it seems almost certain that everyone (except monozygotic twins) will have a unique genotype when considering this entire collection of susceptibility locations. Indeed, we used the 30,248 individuals in the WTCCC dataset to test this hypothesis [13]. The first 102 WTCCC-identified non-*MHC SNPs* [13] were ordered by the strength of their MS-association (i.e., by the magnitude of their respective *ORs*). Considering heterozygotes and homozygotes to be separate genotypes and considering only 20 of the strongest MS-associated haplotypes – together with the (*H*+) genotype in the Class II region – no genotype (including both cases or controls) had more than 2 representations in the WTCCC and considering just 85 of the 103 regions (including *H*+), everyone had a unique genotype. Similarly, considering heterozygotes and homozygotes to be the same genotype and considering only 40 of the 103 regions, no genotype had more than two representations in the WTCCC and considering just 86 regions, everyone had a unique genotype. Clearly, neither including in the analysis the more than 200 loci now-identified MS-associated *SNP*s [14], nor analyzing, at these

susceptibility loci, specific MS-associated *SNP*-haplotypes rather than single *SNP*s [23,24], will alter this conclusion. Everyone in the WTCCC has a unique genotype considering all of their MS-associated loci.

We also analyzed the WTCCC data for every possible combination of three "risk" haplotypes at these 102 locations, together with the state of the (*H*+)-genotype, with regard to their MS-association. There were 960 3-locus combinations (either homozygous or heterozygous), together with a heterozygous state at the (*H*+) locus, that had an *OR*, which significantly exceeded that found by considering the (*H*+) locus by itself. By contrast, there were 7009 such combinations, together with a homozygous state at the (*H*+) locus, that had an *OR*, which significantly exceeded that when considering the (*H*+) locus by itself. Nevertheless, in both circumstances, there was little consistency. Counting the number of cases having each 4-locus combination for heterozygous (*H*+) individuals, yielded: (mean = 51; range: 14 – 582) and, for homozygous (*H*+) individuals, it yielded: (mean = 112; range: 22 – 338). Also, these estimates steadily decreased with each additional locus included in the combination. These observations become more striking when one considers just the 100 most significant MS-associated combinations for individuals heterozygous or homozygous for (*H*+) separately. In the heterozygous group, the number of cases having each combination is: (mean = 34; range: 16 – 92), whereas the number of cases <u>not</u> having each combination is far greater: (mean = 4,272; range: 4,684 – 4,760). Similarly, in the homozygous group, the number of cases having each combination is: (mean = 54; range: 31 – 130), whereas the number of cases <u>not</u> having each combination is, again, far greater: (mean = 753; range: 677 – 776). Thus, it seems clear that, although certain combinations increase the likelihood of (*G*) subset membership, the actual combinations that do this are quite heterogeneous, and only a small proportion of genetically susceptible individuals (who actually develop MS) share even the same 4-locus genetic combination. This finding seems to indicate that genetic susceptibility to MS is largely idiosyncratic.
