# Supplementary material for: The nature of genetic and environmental susceptibility to multiple sclerosis
Source: PLoS One. 2021 Mar 22;16(3):e0246157. doi: 10.1371/journal.pone.0246157 (PMC7984655; doi:10.1371/journal.pone.0246157)
Supplement: Attachment — Submitted filename: Response Letter.doc [file pone.0246157.s002.doc]

# UNIVERSITY OF CALIFORNIA, SAN FRANCISCO

| BERKELEY · DAVIS · IRVINE · LOS ANGELES · MERCED · RIVERSIDE · SAN DIEGO · SANTA CRUZ |  | SANTA BARBARA · SAN FRANCISCO |
| --- | --- | --- |

| Douglas S. Goodin, MD  Professor of Neurology | Department of Neurology, Rm. M794  San Francisco, CA 94143-0114  Tel: (415) 514-2464 Fax: (415) 514-2470  E-mail: douglas.goodin@ucsf.edu |
| --- | --- |
|  |  |

10 January 2021

Sreeram V. Ramagopalan

Academic Editor

PLOS One

Dear Dr. Ramagopalan:

Re:	The Nature of Genetic and Environmental Susceptibility to Multiple Sclerosis

Goodin DS, Khankhanian P, Gourraud PA, Vince N

PONE-D-20-25332

Thank you very much for your letter of 30 December 2020 regarding the above referenced manuscript. Enclosed please find a new version of this manuscript, which has been revised in accordance with the Reviewers comments. I hope that, with this revision, you will now find the manuscript suitable for publication in *PLoS One*. Specifically, we have made the following changes to address the Reviewers concerns:

**Reviewer #1:**

*1. The following sentence may need some additional attention; if indeed EBV infection is present in all MS patients, this can be designated as a 'necessary factor' in the MS ethology. Whether or not the authors are willing to postulate this is important here, if the authors do not state this, this sentence needs rewriting and toning down.
"In addition, a prior Epstein Barr viral (EBV) infection seems to be a prerequisite for most (or all) genotypes in (G) to develop MS [3,4,49,50,60-62]. Indeed, if (as suggested by these studies) a prior EBV infection occurs in 100% of MS cases, this would indicate that EBV exposure is part of the causal pathway leading to MS and that, at least, this environmental exposure is required for disease pathogenesis [49]."*

**Response:** As requested, we have now indicated that, in this circumstance, EBV infection can be designated as a ‘necessary factor’ (p. 26).

*2. For the sake of understanding where the number 0.99 comes from please change the sentence: “"For example, in HIV, if homozygous Δ-32 mutations were completely protective, then: P(G) = 0.99 .*

*To: "For example, in HIV, if homozygous Δ-32 mutations (occurring in 1% of the population) were completely protective, then: P(G) = 0.99 .”*

*And similarly
"By contrast, in SCD, where: P(G) = 0.03 , we would characterize carrying homozygous HbS mutations as the defining trait for membership in the “genetically susceptible” subset."
To "By contrast, in SCD, where: P(G) = 0.03 , we would characterize carrying homozygous HbS mutations (3% of individuals) as the defining trait for membership in the “genetically susceptible” subset."*

**Response:** We have now modified these sentences as suggested (p. 10)

*3. The authors state that at tiny fraction of the population is genetically susceptible and refer to the number of less than 4,7%; This is not a ‘tiny’ fraction, and could rather be described as a merely ‘fraction’*

**Response:** We have now modified this sentence as suggested (p. 35)

**Reviewer #2:**

1. *For readers who are not familiar with IMSGC study (Science 2019, 365:eeav7188), rather than “over 200 genes” in the Abstract and Introduction, it might be worth saying that based on SNP data there are currently 233 genes associated with MS susceptibility, including 32 genes within the MHC, and the first locus identified on a sex chromosome, on the X chromosome. The SNPs are located within or near to immune related genes, and implicate both the adaptive and innate arms of the immune system. The MHC DRB1*15:01-DQB1*06:02 haplotype has the strongest association, with about a 3-fold increased risk of MS. About 23% of the general population in Europe and North America carries this haplotype, but 80% of this group are not at risk of MS.*

**Response:** As suggested, we have now incorporated these important points into our *Abstract* and *Introduction* (p.3 & p.6).

*2. Regarding life expectancy of people with MS, p.15 and references #37-41 dating from 2008-2014, I think it is worth pointing out that life expectancy has been improving, e.g. Koch-Henriksen N, et al. J Neurol Neurosurg Psychiatry 2017;88:626–631.*

**Response:** We agree and have now referenced this paper and added a comment regarding these findings (p.11).

*3. The prevalence data cited on p.16, references #42 and #43 date from 1997 to 2001. There is more recent data from Wallin MT et al 2019, Neurology March 5, 92(10): e1-e12, which estimates the prevalence of MS in the US fat between 337.9 per 100,000 population (n = 851,749 persons with MS), to 362.6 per 100,000 population (n = 913,925 persons with MS).*

**Response:** We agree that this is an important study needs to be referenced. We have now included a reference to, and a brief discussion of, their findings (p.11).

*4. The reference cited in the legend to Fig 3, I think should be #54 Orton et al 2006, rather than reference #58.*

**Response:** The Reviewer is correct. This has now been changed (Fig 3).

*5. I think the statement that “MS is fundamentally a genetic disorder” is perhaps too strong. The point being made is that only a small proportion of the population is at risk for developing MS. In the Introduction and Discussion, the authors note that susceptibility to MS involves both environmental and genetic factors. There are few diseases which are purely genetic or purely environmental, and there is an interaction between genes and environment to varying degrees. MS cannot develop without the right environmental exposures. The paper takes the position that genetics makes the predominant contribution.*

**Response:** We agree completely with the Reviewer. Our statement incorrectly implied that genetics was of predominant importance and we have now modified it to make the point that both genetics and the environment are required (p.35).

*6. The paper is not an easy read. There is a fairly long section in the middle in which it is possible to become lost in the mathematical symbols, and the need for reference back to the symbols or to Table 1. I think this detracts from the flow of the paper and the key ideas proposed. Perhaps more of the statistical and mathematical treatment could be in the supplementary section. The formula with an explanation in words in the text, with the derivation of the formula in a supplementary section or footnote, might be easier to follow.*

**Response:** We appreciate the fact that this paper is somewhat difficult to read. We have now been over the manuscript and tried to make it more clear. We have also relaxed the requirement that, when the penetrance values of subsets (G1) and (G2) are different, the distribution of each are unimodal. This change has slightly increased our upper estimate of P(G) for Lower Solutions. We have also reduced the mathematics in the Main Text and moved some of this to the Supplemental Material. However, I suspect that the Reviewer is referring mostly to our presentations both of Proposition #1 and also of the environmental impacts on MS pathogenesis (Section #7). We feel that these particular sections are so central to our conclusions that we are very reluctant to move them. Nevertheless, we leave this to your editorial discretion.

I hope that, with these modifications and additions, you will now find the manuscript suitable for publication in *PLoS One*. Thank you very much for your consideration of this matter. I look forward to hearing from you in due course.

Yours sincerely,

Douglas S. Goodin, MD

*Professor of Neurology*

*University of California, San Francisco*
